# Cytokeratin and Neuroendocrine Positivity in Cutaneous Small Blue Round Cell Tumor—Is It Always Merkel Cell Carcinoma?

**DOI:** 10.1111/cup.14844

**Published:** 2025-07-10

**Authors:** Simon Moubarak, John McAfee, Karen Fritchie, Jennifer S. Ko, Steven D. Billings, Shira Ronen

**Affiliations:** ^1^ Department of Pathology Cleveland Clinic Cleveland Ohio USA

**Keywords:** cutaneous Ewing sarcoma, cytokeratin markers, Merkel cell carcinoma, neuroendocrine markers

## Abstract

We present a case of a 39‐year‐old woman initially diagnosed with Merkel cell carcinoma (MCC), a highly aggressive neuroendocrine carcinoma, due to the presence of cytokeratin and neuroendocrine marker expression. The tumor was dermal based, showing small round blue cells with fine chromatin, scant cytoplasm, and scattered mitotic figures arranged in sheets, small cohesive nests, and cords within sclerotic to edematous stroma. Provided immunohistochemical stains showed strong pancytokeratin expression coupled with perinuclear dot‐like staining for cytokeratin 20 in a distinct regional distribution, predominantly in areas where the tumor cells formed cohesive nests and cords within sclerotic stroma. Stains for neuroendocrine markers, including synaptophysin, INSM1, and CD56, were positive, albeit focal or regional in the more cohesive areas. Given the patient's age and unusual regional staining patterns, additional testing was performed, which revealed diffuse membranous CD99 staining and *EWSR1::ERG* fusion. These findings led us to revise the diagnosis to cutaneous Ewing sarcoma (ES). The distinction between MCC and cutaneous ES is crucial due to their different survival rates and treatment approaches. This case underscored the importance of considering alternative diagnoses when encountering cutaneous small round blue cell tumors in the presence of unusual histologic and immunohistochemical findings, particularly in younger patients.

## 1 Introduction

Merkel cell carcinoma (MCC) is an aggressive neuroendocrine cancer that predominantly affects sun‐exposed areas of the skin, such as the head and neck, extremities, and trunk, particularly in elderly individuals. Histologically, it is characterized by a dermal or subcutaneous‐based proliferation of small, round‐to‐ovoid blue cells with a fine stippled chromatin pattern [1, 2, 3]. Ewing sarcoma (ES) is another aggressive, small, round blue cell tumor with a high nuclear‐to‐cytoplasmic ratio and fine chromatin. However, it is most common in children and young adults, arising both in bone and soft tissue [4]. The occurrence of cutaneous ES, often referred to as primary superficial ES, is relatively rare. It has been observed to differ significantly in terms of epidemiology and prognosis from conventional ES. Multiple case series have shown that superficial ES behaves far less aggressively and has a more favorable prognosis compared to its deep‐seated counterpart [5, 6, 7]. Furthermore, there is considerable variation in the respective treatment approaches of ES and MCC [8, 9].

Aside from the clear histologic similarities between MCC and ES, the distinction between the two entities becomes more challenging due to overlap in their immunohistochemical profiles, as ES has been reported to express cytokeratins as well as markers of neuroendocrine differentiation in certain cases [10, 11]. We present a case of cutaneous ES, which was initially misdiagnosed as MCC due to aberrant expression of cytokeratin and neuroendocrine markers. Advanced molecular studies confirmed the presence of *EWSR1::ERG* fusion.

## 2 Case Report

**FIGURE 1:**
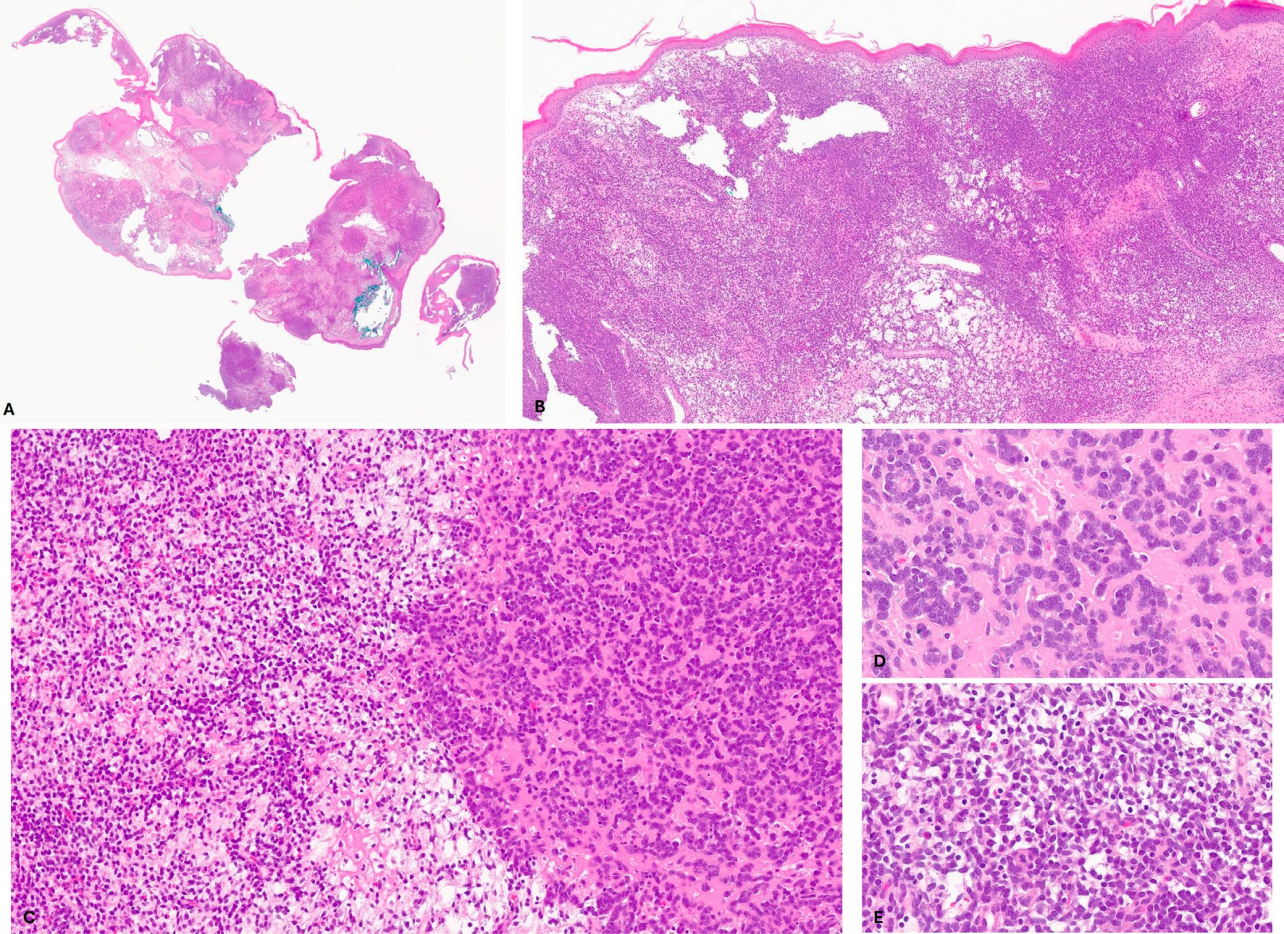
(A) Scanning magnification of the tumor showed multiple sections of polypoid tumor (H&E, scanning magnification). (B) The tumor involved the dermis comprising sheets, small cohesive nests, and cords of cells (H&E, ×30). (C) The neoplastic cells were embedded in a sclerotic to edematous stroma (H&E, ×120). (D,E) The lesional cells exhibited small round to ovoid nuclei, fine chromatin, variably small nucleoli, and scant eosinophilic to clear cytoplasm (H&E, ×400).

We received a referral case of a 39‐year‐old woman who presented with a growing lesion on her right breast over the last 6 months. Histologic sections demonstrated a dermal polypoid tumor, comprising sheets, small cohesive nests, and cords of uniform round cells embedded in a sclerotic to edematous stroma (Figure 1A–C). The lesional cells exhibited small round to ovoid nuclei, fine chromatin, variably small nucleoli, and scant eosinophilic to clear cytoplasm (Figure 1D,E). There were scattered mitotic figures, mainly in the sclerotic areas. The overlying epidermis was thin and displayed mild spongiosis.

**FIGURE 2:**
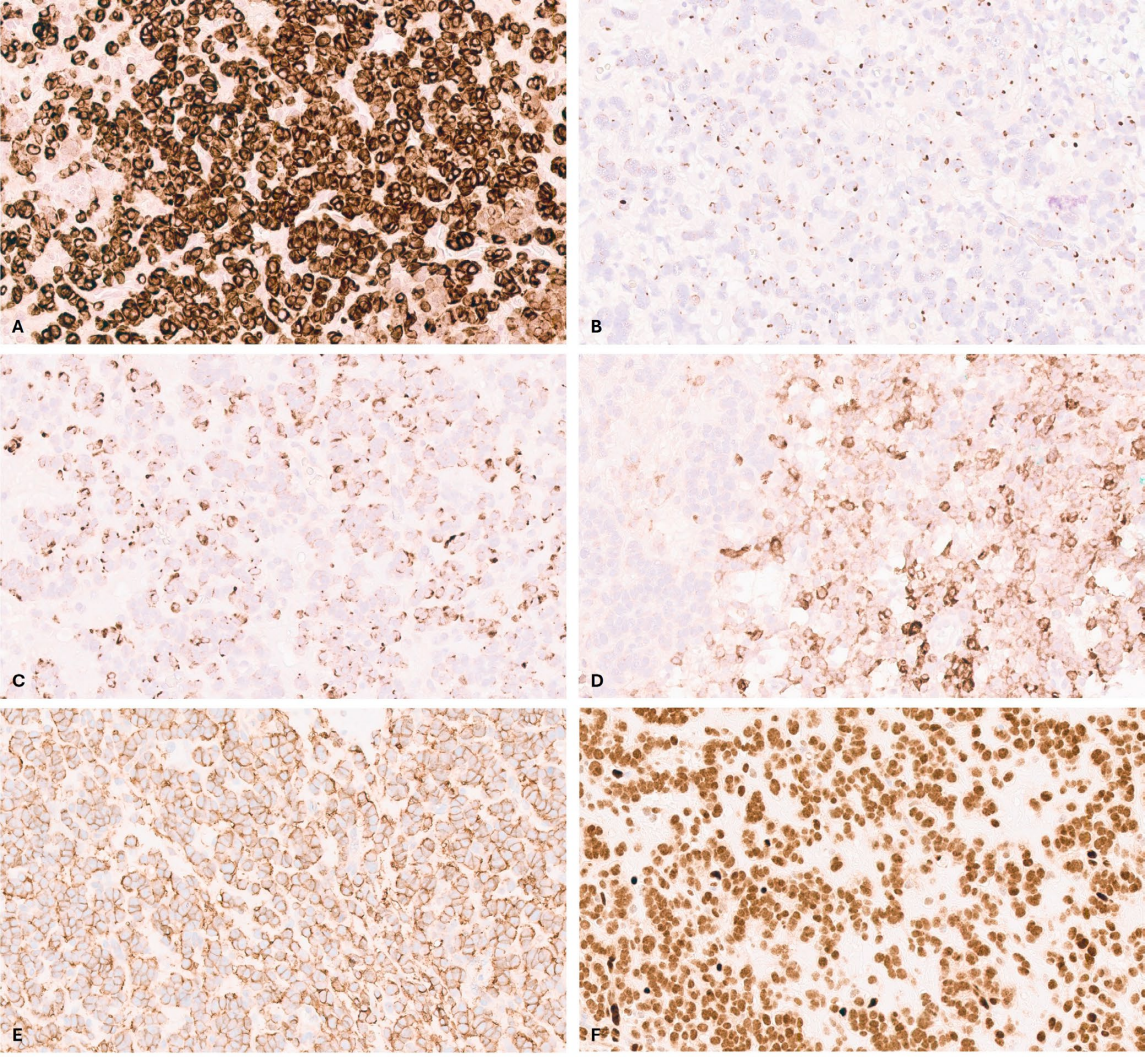
Immunohistochemical profile of the case. (A) The tumor showed strong pancytokeratin staining in a distinct regional distribution, predominantly in areas within the sclerotic stroma (IHC, ×400). (B,C) CK20 and CAM5.2 immunohistochemical stains were also positive in areas showing perinuclear dot‐like pattern, largely in the same regions where pancytokeratin was positive (IHC, ×400). (D) Synaptophysin immunohistochemical stain was focally positive, mainly in the more cohesive areas (IHC, ×400). (E) The neoplastic cells displayed diffuse membranous CD99 staining (IHC, ×400). (F) Immunohistochemical stain for ERG demonstrated diffuse nuclear positivity within the tumor cells (IHC, ×400).

Immunohistochemically, the tumor showed strong pancytokeratin staining in a distinct regional distribution, predominantly in areas where the tumor cells formed cohesive nests and cords within sclerotic stroma (Figure 2A). CAM5.2 and CK20 stains were also positive in a perinuclear dot‐like pattern, largely in the same regions where pancytokeratin was positive (Figure 2B,C). Stains for neuroendocrine markers, including synaptophysin, INSM1, and CD56, were positive, albeit focally, in the more cohesive areas (Figure 2D). The neoplastic cells were negative for CK7, CK5, CD34, TTF1, chromogranin, p40, CD45, CD3, CD20, myeloperoxidase, and GATA3. The original referral pathologist diagnosed this tumor as MCC based on the morphology and immunohistochemical profile.

Due to the unusual regional staining in various areas, we expanded the immunohistochemical panel to further characterize the lesion. The tumor cells displayed diffuse membranous CD99 staining and nuclear positivity for ERG (Figure 2E,F). The cells lacked expression of SOX10, EMA, desmin, Merkel cell polyomavirus (MCPyV), DUX4, SSX, and SS18‐SSX.

Given the presence of diffuse CD99 positivity in a membranous distribution coupled with nuclear ERG positivity, we considered the possibility of a fusion‐associated soft tissue tumor. To further explore this possibility, we performed a next‐generation sequencing panel for sarcoma‐associated gene fusions, which detected an *EWSR1::ERG* gene rearrangement.

Overall, given the histologic features, immunohistochemical profile, and molecular findings, this lesion was best characterized as a cutaneous ES. A re‐excision of the lesion was negative for residual tumor. PET CT scan showed no evidence of distal metastasis. The patient was recommended to begin adjuvant chemotherapy.

## 3 Discussion

The diagnosis of small round blue cell tumors can be challenging due to their broad differential diagnosis, which includes neuroblastoma, neuroendocrine carcinoma, lymphoma, melanoma, and primitive‐appearing sarcomas such as alveolar rhabdomyosarcoma, poorly differentiated synovial sarcoma, ES, and *CIC‐*rearranged sarcoma [12, 13, 14]. Factors such as the location of the lesion and the patient demographics may provide useful clues to differentiate these diagnoses. While immunohistochemical stains may yield helpful ancillary data, overlapping immunohistochemical profiles of these entities can sometimes complicate this process [10, 11, 15]. We present a case of a 39‐year‐old female who was referred to our institution for further treatment with a diagnosis of MCC of the breast. However, upon reviewing the material, using additional immunohistochemical stains and molecular testing, the diagnosis was revised to ES.

MCC, which most commonly arises on sun‐damaged skin of elderly males, would be an unusual diagnosis in a young adult female patient [16]. Secondly, although MCC may be composed of epithelioid to round cells with finely dispersed chromatin and inconspicuous nucleoli, the relative monotony of the neoplastic population in this case gave us pause. MCCs are classically positive for epithelial and neuroendocrine markers; however, the tumor cells characteristically show strong and diffuse expression of these immunostains. Specifically, MCC typically exhibits CK20 staining in a perinuclear/paranuclear dot‐like pattern in most of the tumor cells. Yet, our tumor only demonstrated focal CK20 staining [17, 18, 19, 20]. While most MCC show strong staining with neuroendocrine markers [21, 22, 23, 24, 25], our tumor only exhibited regional focal staining for synaptophysin, INSM1, and CD56 and lacked chromogranin expression entirely. The inconsistent staining distribution of this immunopanel led to the consideration of malignancies that may show aberrant expression of these markers rather than be defined by them [4, 14].

Most MCC cases worldwide are caused by the integration of the MCPyV, while the remaining cases are due to ultraviolet‐induced damage to the tumor genome. MCPyV‐negative MCCs have a higher mutational burden compared to those associated with the virus. They are also associated with multiple mutations such as TERT promoter, TP53, and RB1 mutations. A recent case report also described the first MCPyV‐associated MCC case to have an *NSD3::FGFR1* fusion [26]. The presence of MCPyV immunochemistry helps establish the association of MCC with the virus. However, the reported immunohistochemical expression of the MCPyV large T‐antigen varies significantly, ranging from 25% to 90% of MCC cases [27, 28]. The variability could be attributed to differences in detection sensitivity and geographical variations in tumor etiology. In our case, the absence of MCPyV did not provide conclusive evidence of virus association with the tumor.

These combined features led us to broaden our differential diagnosis to include round cell sarcomas. Given the uniformity of the neoplastic component combined with the young age of the patient, we focused our work‐up on fusion‐driven sarcomas. The absence of myogenic markers helped to exclude alveolar rhabdomyosarcoma, while ‘next‐generation immunohistochemistry’, which exploits the molecular genetics of mesenchymal neoplasms, including SS18‐SSX and DUX4, helped to rule out synovial sarcoma and *CIC*‐rearranged sarcomas, respectively. Ultimately, the characteristic pattern of CD99 staining and ERG positivity in an otherwise undifferentiated round cell sarcoma led to the consideration of ES, which was confirmed by molecular studies showing *EWSR1::ERG* fusion. CD99 staining may be appreciated in MCC, but often appears as a dot‐like pattern rather than membranous and diffuse [2, 7, 10, 11, 14, 29, 30, 31, 32]. Additionally, several studies have demonstrated that ERG immunohistochemistry can serve as a surrogate marker for detecting ERG rearrangements in Ewing sarcomas I. Notably, Wei‐Lien Wang et al. reported that antibodies targeting the C‐terminus of ERG exhibit 88% sensitivity and 88% specificity for identifying ERG rearrangements in Ewing sarcomas harboring the *EWSR1::ERG* fusion [33].

A subset of mesenchymal neoplasia is definitionally cytokeratin‐positive, including epithelioid sarcoma, synovial sarcoma, and the recently recognized *TFCP2*‐rearranged spindled and epithelioid rhabdomyosarcoma [34, 35, 36]. Conversely, numerous sarcomas, such as ES and leiomyosarcoma, may show aberrant expression of various cytokeratins. ES has been reported to display cytokeratin positivity in a subset of cases, with the dot‐like keratin staining pattern occurring in up to 10% of ES. This pattern, however, is usually focal, as seen in our case where staining was predominant in the more cohesive areas [7, 10]. Similarly, sarcomas may also show aberrant expression of neuroendocrine markers, even chromogranin; however, neuroendocrine marker expression in ES in particular is not well documented in the literature [37].

Given the immense degree of overlap of morphologic and immunohistochemical profiles of neuroendocrine carcinomas and specific sarcomas, molecular genetics played a crucial role in our diagnosis. Even though it has been recently recognized that *FET::ETS* fusions are not unique to ES, to date, these fusions have been identified in MCC thus far [38]. Instead, MCC is typically characterized by point mutations or copy number alterations involving tumor suppressor genes such as *TP53* and *RB1* [39, 40]. Additionally, MCC usually exhibits either integration of the MCPyV or a UV mutational signature [41]. Furthermore, it is notable that, despite its rarity, ERG gene rearrangements, including ERG fusions, have been described in ES cases [42]. *FET::ETS* fusions, including *EWSR1::ERG* have also been reported in the newly emerging superficial neurocristic *FET::ETS* fusion tumor. However, these tumors exhibit diffuse SOX10 and S100 protein expression, whereas our tumor was negative for SOX10.

The distinction between MCC and ES is crucial as each has unique survival rates and treatment approaches. The 5‐year survival for MCC patients is estimated at 51% for patients with localized disease, 35% for those with regional metastasis, and 14% for patients with distant metastasis [43]. Conversely, patients with cutaneous and superficial ES exhibit an overall survival rate of 93%, and the estimated 10‐year survival probability is approximately 90% [44]. MCC is a radiosensitive tumor, and radiation therapy is a key component in managing the disease locally and regionally [45]. Recent advancements in immunomodulatory therapies have also shown potential in improving survival rates for patients with advanced stages of the disease. On the other hand, recent studies on the treatment of cutaneous and subcutaneous ES reveal a high rate of local control, a low rate of metastatic disease, and an excellent overall outcome with less intensive chemotherapy, as well as personalized treatment plans to reduce or avoid radiation therapy in completely resected cases.

In conclusion, not every cutaneous small round blue cell tumor that expresses cytokeratins in a perinuclear dot‐like pattern in conjunction with neuroendocrine markers is MCC. Other diagnoses, such as cutaneous ES, should be considered, especially in younger patients with unusual histologic or immunohistochemical findings.

## Conflicts of Interest

The authors declare no conflicts of interest.

## Data Availability

The data that support the findings of this study are available from the corresponding author upon reasonable request.

## References

[1] D. Schadendorf , C. Lebbé , A. zur Hausen , et al., “Merkel Cell Carcinoma: Epidemiology, Prognosis, Therapy and Unmet Medical Needs,” European Journal of Cancer 71 (2017): 53–69, 10.1016/j.ejca.2016.10.022. 27984768

[2] T. Kervarrec , A. Tallet , E. Miquelestorena‐Standley , et al., “Diagnostic Accuracy of a Panel of Immunohistochemical and Molecular Markers to Distinguish Merkel Cell Carcinoma From Other Neuroendocrine Carcinomas,” Modern Pathology 32, no. 4 (2019): 499–510, 10.1038/s41379-018-0155-y. 30349028

[3] T. Ogawa , P. Donizy , C. L. Wu , K. M. Cornejo , J. Ryś , and M. P. Hoang , “Morphologic Diversity of Merkel Cell Carcinoma,” American Journal of Dermatopathology 42, no. 9 (2020): 629–640, 10.1097/DAD.0000000000001548. 32833736

[4] H. T. Wu and D. Govender , “Ewing Sarcoma Family of Tumours: Unusual Histological Variants and Immunophenotypic Characteristics,” Diagnostic Histopathology 18, no. 8 (2012): 348–355, 10.1016/j.mpdhp.2012.06.006.

[5] L. D. Murphy , G. M. Orman , J. A. Bridge , G. Bajaj , J. M. Gardner , and D. P. Douglass , “Primary Superficial Ewing Sarcoma: A Unique Entity? A Case Report Including Novel Findings of and Copy Number Loss,” Journal of Cutaneous Pathology 47, no. 10 (2020): 970–975, 10.1111/cup.13762. 32483824

[6] A. B. I. Collier , L. Simpson , and P. Monteleone , “Cutaneous Ewing Sarcoma: Report of 2 Cases and Literature Review of Presentation, Treatment, and Outcome of 76 Other Reported Cases,” Journal of Pediatric Hematology/Oncology 33, no. 8 (2011): 631–634, 10.1097/MPH.0b013e31821b234d. 22042282

[7] S. L. Hasegawa , J. M. Davison , A. Rutten , J. A. Fletcher , and C. D. M. Fletcher , “Primary Cutaneous Ewing's Sarcoma: Immunophenotypic and Molecular Cytogenetic Evaluation of Five Cases,” American Journal of Surgical Pathology 22, no. 3 (1998): 310–318. 9500772 10.1097/00000478-199803000-00005

[8] C. Rodriguez‐Galindo , S. L. Spunt , and A. S. Pappo , “Treatment of Ewing Sarcoma Family of Tumors: Current Status and Outlook for the Future,” Medical and Pediatric Oncology 40, no. 5 (2003): 276–287, 10.1002/mpo.10240. 12652615

[9] W. J. McAfee , C. G. Morris , C. M. Mendenhall , J. W. Werning , N. P. Mendenhall , and W. M. Mendenhall , “Merkel Cell Carcinoma,” Cancer 104, no. 8 (2005): 1761–1764, 10.1002/cncr.21355. 16136596

[10] M. Gu , C. R. Antonescu , G. Guiter , A. G. Huvos , M. Ladanyi , and M. F. Zakowski , “Cytokeratin Immunoreactivity in Ewing's Sarcoma: Prevalence in 50 Cases Confirmed by Molecular Diagnostic Studies,” American Journal of Surgical Pathology 24, no. 3 (2000): 410–416. 10716155 10.1097/00000478-200003000-00010

[11] I. Machado , S. Navarro , J. A. López‐Guerrero , et al., “Neuroendocrine Differentiation in a Large Series of Genetically‐Confirmed Ewing's Sarcoma Family Tumor: Does It Provide Any Diagnostic or Prognostic Information?,” Pathology, Research and Practice 219 (2021): 153362, 10.1016/j.prp.2021.153362. 33610950

[12] T. B. Lewis , C. M. Coffin , and P. S. Bernard , “Differentiating Ewing's Sarcoma From Other Round Blue Cell Tumors Using a RT‐PCR Translocation Panel on Formalin‐Fixed Paraffin‐Embedded Tissues,” Modern Pathology 20, no. 3 (2007): 397–404, 10.1038/modpathol.3800755. 17334332

[13] I. Brčić , T. Brodowicz , L. Cerroni , et al., “Undifferentiated Round Cell Sarcomas With *CIC‐DUX4* Gene Fusion: Expanding the Clinical Spectrum,” Pathology (Philadelphia, Pa.) 52, no. 2 (2020): 236–242, 10.1016/j.pathol.2019.09.015. 31870501

[14] M. Pulitzer , “Merkel Cell Carcinoma,” Surgical Pathology Clinics 10, no. 2 (2017): 399–408, 10.1016/j.path.2017.01.013. 28477888 PMC5443625

[15] N. A. Trikalinos , J. S. A. Chrisinger , and B. A. Van Tine , “Common Pitfalls in Ewing Sarcoma and Desmoplastic Small Round Cell Tumor Diagnosis Seen in a Study of 115 Cases,” Medical Science 9, no. 4 (2021): 62, 10.3390/medsci9040062. PMC8544526 34698236

[16] J. Albores‐Saavedra , K. Batich , F. Chable‐Montero , N. Sagy , A. M. Schwartz , and D. E. Henson , “Merkel Cell Carcinoma Demographics, Morphology, and Survival Based on 3870 Cases: A Population Based Study,” Journal of Cutaneous Pathology 37, no. 1 (2010): 20–27, 10.1111/j.1600-0560.2009.01370.x. 19638070

[17] R. Moll , A. Löwe , J. Laufer , and W. W. Franke , “Cytokeratin 20 in Human Carcinomas. A New Histodiagnostic Marker Detected by Monoclonal Antibodies,” American Journal of Pathology 140, no. 2 (1992): 427–447. 1371204 PMC1886432

[18] M. Miettinen , “Keratin 20: Immunohistochemical Marker for Gastrointestinal, Urothelial, and Merkel Cell Carcinomas,” Modern Pathology 8, no. 4 (1995): 384–388. 7567935

[19] J. K. C. Chan , S. Suster , B. M. Wenig , W. Y. W. Tsang , J. B. K. Chan , and A. L. W. Lau , “Cytokeratin 20 Immunoreactivity Distinguishes Merkel Cell (Primary Cutaneous Neuroendocrine) Carcinomas and Salivary Gland Small Cell Carcinomas From Small Cell Carcinomas of Various Sites,” American Journal of Surgical Pathology 21, no. 2 (1997): 226–234. 9042291 10.1097/00000478-199702000-00014

[20] A. J. Hanly , G. W. Elgart , M. Jorda , J. Smith , and M. Nadji , “Analysis of Thyroid Transcription Factor‐1 and Cytokeratin 20 Separates Merkel Cell Carcinoma From Small Cell Carcinoma of Lung,” Journal of Cutaneous Pathology 27, no. 3 (2000): 118–120, 10.1034/j.1600-0560.2000.027003118.x. 10728812

[21] E. Haneke , H. J. Schulze , and G. Mahrle , “Immunohistochemical and Immunoelectron Microscopic Demonstration of Chromogranin A in Formalin‐Fixed Tissue of Merkel Cell Carcinoma,” Journal of the American Academy of Dermatology 28, no. 2 (1993): 222–226, 10.1016/0190-9622(93)70031-N. 8432919

[22] V. Koljonen , C. Haglund , E. Tukiainen , and T. Böhling , “Neuroendocrine Differentiation in Primary Merkel Cell Carcinoma ‐ Possible Prognostic Significance,” Anticancer Research 25, no. 2A (2005): 853–858. 15868919

[23] M. Kurokawa , K. Nabeshima , Y. Akiyama , et al., “CD56: A Useful Marker for Diagnosing Merkel Cell Carcinoma,” Journal of Dermatological Science 31, no. 3 (2003): 219–224, 10.1016/S0923-1811(03)00029-X. 12727026

[24] M. T. Lilo , Y. Chen , and R. E. LeBlanc , “INSM1 Is More Sensitive and Interpretable Than Conventional Immunohistochemical Stains Used to Diagnose Merkel Cell Carcinoma,” American Journal of Surgical Pathology 42, no. 11 (2018): 1541–1548, 10.1097/PAS.0000000000001136. 30080705

[25] P. S. Rush , J. N. Rosenbaum , M. Roy , R. M. Baus , D. D. Bennett , and R. V. Lloyd , “Insulinoma‐Associated 1: A Novel Nuclear Marker in Merkel Cell Carcinoma (Cutaneous Neuroendocrine Carcinoma),” Journal of Cutaneous Pathology 45, no. 2 (2018): 129–135, 10.1111/cup.13079. 29148079

[26] V. Lenskaya , R. K. Yang , P. P. Aung , V. G. Prieto , P. Nagarajan , and W. C. Cho , “NSD3::FGFR1: A Novel Gene Fusion First to be Described in Merkel Cell Carcinoma,” American Journal of Dermatopathology 47, no. 5 (2022): 400–403, 10.1097/DAD.0000000000002953. 40036479

[27] A. G. Miner , R. M. Patel , D. A. Wilson , et al., “Cytokeratin 20‐Negative Merkel Cell Carcinoma Is Infrequently Associated With the Merkel Cell Polyomavirus,” Modern Pathology 28, no. 4 (2015): 498–504, 10.1038/modpathol.2014.148. 25394777

[28] M. Dabner , R. J. McClure , N. T. Harvey , et al., “Merkel Cell Polyomavirus and p63 Status in Merkel Cell Carcinoma by Immunohistocnemistry: Merkel Cell Polyomavirus Positivity Is Inversely Correlated With Sun Damage, but Neither Is Correlated With Outcome,” Pathology (Philadelphia, Pa.) 46, no. 3 (2014): 205–210, 10.1097/PAT.0000000000000069. 24614722

[29] S. H. Olsen , D. G. Thomas , and D. R. Lucas , “Cluster Analysis of Immunohistochemical Profiles in Synovial Sarcoma, Malignant Peripheral Nerve Sheath Tumor, and Ewing Sarcoma,” Modern Pathology 19, no. 5 (2006): 659–668, 10.1038/modpathol.3800569. 16528378

[30] T. Kervarrec , A. Tallet , E. Miquelestorena‐Standley , et al., “Morphologic and Immunophenotypical Features Distinguishing Merkel Cell Polyomavirus‐Positive and Negative Merkel Cell Carcinoma,” Modern Pathology 32, no. 11 (2019): 1605–1616, 10.1038/s41379-019-0288-7. 31201352

[31] S. A. Nicholson , M. B. McDermott , P. E. Swanson , and M. R. Wick , “CD99 and Cytokeratin‐20 in Small‐Cell and Basaloid Tumors of the Skin,” Applied Immunohistochemistry & Molecular Morphology 8, no. 1 (2000): 37–41. 10937047 10.1097/00129039-200003000-00006

[32] A. Rajagopalan , D. Browning , and S. Salama , “CD99 Expression in Merkel Cell Carcinoma: A Case Series With an Unusual Paranuclear Dot‐Like Staining Pattern,” Journal of Cutaneous Pathology 40, no. 1 (2013): 19–24, 10.1111/cup.12049. 23145531

[33] W. L. Wang , N. R. Patel , M. Caragea , et al., “Expression of ERG, an Ets Family Transcription Factor, Identifies ERG‐Rearranged Ewing Sarcoma,” Modern Pathology 25, no. 10 (2012): 1378–1383, 10.1038/modpathol.2012.97. 22766791

[34] V. Andrei , S. Haefliger , and D. Baumhoer , “Superficial Mesenchymal Tumours Expressing Epithelial Markers on Immunohistochemistry: Diagnostic Clues and Pitfalls,” Seminars in Diagnostic Pathology 40, no. 4 (2023): 238–245, 10.1053/j.semdp.2023.04.016. 37147159

[35] I. Machado , E. Wardelmann , M. Zhao , et al., “Primary Cutaneous Rhabdomyosarcoma With EWSR1/FUS::TFCP2 Fusion: Four New Cases With Distinctive Morphology, Immunophenotypic, and Genetic Profile,” Virchows Archiv 486 (2024): 1187–1198, 10.1007/s00428-024-04007-z. 39692858

[36] J. L. Silva Cunha , I. L. Cavalcante , C. C. da Silva Barros , et al., “Intraosseous Rhabdomyosarcoma of the Maxilla With TFCP2 Fusion: A Rare Aggressive Subtype With Predilection for the Gnathic Bones,” Oral Oncology 130 (2022): 105876, 10.1016/j.oraloncology.2022.105876. 35550988

[37] A. Bahrami , A. M. Gown , G. S. Baird , M. J. Hicks , and A. L. Folpe , “Aberrant Expression of Epithelial and Neuroendocrine Markers in Alveolar Rhabdomyosarcoma: A Potentially Serious Diagnostic Pitfall,” Modern Pathology 21, no. 7 (2008): 795–806, 10.1038/modpathol.2008.86. 18487991

[38] C. A. Dehner , L. M. Warmke , B. Umphress , et al., “Superficial Neurocristic FET::ETS Fusion Tumor: Expanding the Clinicopathological and Molecular Genetic Spectrum of a Recently Described Entity,” Modern Pathology 38, no. 2 (2025): 100656, 10.1016/j.modpat.2024.100656. 39522640

[39] C. Stheneur , L. Faivre , G. Collod‐Beroud , et al., “Prognosis Factors in Probands With an FBN1 Mutation Diagnosed Before the Age of 1 Year,” Pediatric Research 69, no. 3 (2011): 265–270, 10.1203/PDR.0b013e3182097219. 21135753

[40] K. J. Fritchie , B. Ameline , V. Andrei , et al., “DNA Methylation Profiling Distinguishes Adamantinoma‐Like Ewing Sarcoma From Conventional Ewing Sarcoma,” Modern Pathology 36, no. 11 (2023): 100301, 10.1016/j.modpat.2023.100301. 37567448 PMC11195538

[41] P. W. Harms , P. Vats , M. E. Verhaegen , et al., “The Distinctive Mutational Spectra of Polyomavirus‐Negative Merkel Cell Carcinoma,” Cancer Research 75, no. 18 (2015): 3720–3727, 10.1158/0008-5472.CAN-15-0702. 26238782 PMC4573907

[42] S. Chen , K. Deniz , Y. S. Sung , L. Zhang , S. Dry , and C. R. Antonescu , “Ewing Sarcoma With ERG Gene Rearrangements: A Molecular Study Focusing on the Prevalence of FUS‐ERG and Common Pitfalls in Detecting EWSR1‐ERG Fusions by FISH,” Genes, Chromosomes & Cancer 55, no. 4 (2015): 340, 10.1002/gcc.22336. 26690869 PMC5006947

[43] K. L. Harms , M. A. Healy , P. Nghiem , et al., “Analysis of Prognostic Factors From 9,387 Merkel Cell Carcinoma Cases Forms the Basis for the New 8th Edition AJCC Staging System,” Annals of Surgical Oncology 23, no. 11 (2016): 3564–3571, 10.1245/s10434-016-5266-4. 27198511 PMC8881989

[44] M. Delaplace , C. Lhommet , G. de Pinieux , B. Vergier , A. de Muret , and L. Machet , “Primary Cutaneous Ewing Sarcoma: A Systematic Review Focused on Treatment and Outcome,” British Journal of Dermatology 166, no. 4 (2012): 721–726, 10.1111/j.1365-2133.2011.10743.x. 22098102

[45] J. A. Vargo , E. R. Ghareeb , G. K. Balasubramani , and S. Beriwal , “RE: Adjuvant Radiation Therapy and Chemotherapy in Merkel Cell Carcinoma: Survival Analyses of 6908 Cases From the National Cancer Data Base,” Journal of the National Cancer Institute 109, no. 10 (2017): djx052, 10.1093/jnci/djx052. 28423400

